# Migraine With Aura Accompanied by Myoclonus: A Case Report

**DOI:** 10.7759/cureus.69046

**Published:** 2024-09-09

**Authors:** Koji Hayashi, Asuka Suzuki, Yuka Nakaya, Naoko Takaku, Toyoaki Miura, Mamiko Sato, Yasutaka Kobayashi

**Affiliations:** 1 Department of Rehabilitation Medicine, Fukui General Hospital, Fukui, JPN; 2 Graduate School of Health Science, Fukui Health Science University, Fukui, JPN

**Keywords:** involuntary limb movement, migraine, migraine headaches, migraine with aura, myoclonus

## Abstract

Migraine is a condition characterized by pulsating headaches, often accompanied by photophobia, phonophobia, and/or gastrointestinal symptoms such as nausea and vomiting. Approximately 15% to one-third of migraine patients experience an aura either before or during the headache. To the best of our knowledge, the occurrence of migraine with myoclonus is extremely rare. This report describes a rare case of migraine with aura accompanied by myoclonus. The patient is a 46-year-old man who developed a visual aura followed by vomiting and a throbbing headache on the right side. As the headache intensified, involuntary movements of the left lower extremity appeared. Brain magnetic resonance imaging (MRI) revealed no structural abnormalities or stroke lesions; however, arterial spin labeling MRI showed hypoperfusion in the right cerebral hemisphere. An ophthalmological evaluation was unremarkable. He was diagnosed with migraine with myoclonus, and the intravenous administration of diazepam and sumatriptan resulted in the cessation of the myoclonus and mild relief of the headache. By the day after admission, the myoclonus and visual symptoms had completely disappeared. The headache resolved by the third day of admission.

## Introduction

Migraine is a complex condition characterized by a pulsating headache, typically localized to one side of the head, with moderate to severe intensity, lasting from four to 72 hours, and often accompanied by photophobia, phonophobia, and/or digestive symptoms such as nausea and vomiting [1]. In about 15% to one-third of migraine sufferers, the headache is preceded or accompanied by an aura, which consists of reversible focal neurological symptoms that can be visual, sensory, speech-related, and/or motor-related [1]. These symptoms develop gradually, spread, and then dissipate [1]. In this report, we describe a rare case of migraine with aura followed by myoclonus.

## Case presentation

A previously healthy 46-year-old man with a history of concussion at the age of 15 presented with visual symptoms in both eyes, followed by vomiting and a throbbing headache on the right side. As the headache intensified, involuntary movements of the left lower extremity appeared. Regarding the visual symptoms, he reported experiencing pixelated vision, a sensation of the vision moving, and scotoma in the bilateral eyes. The involuntary movements involved rapid, rhythmic, small contractions of the muscle groups in the left thigh and lower leg, which repeated continuously. Sometimes the involuntary movements stopped but soon reoccurred. The patient made efforts to stop the movements but was unable to do so and complained of discomfort. He had no reported history of abnormal growth or development and no family history of migraines. He had a history of visual disturbances followed by headaches once every six months to a year, but this episode was the first time he had experienced involuntary movements. He presented with persistent bilateral visual symptoms, headache, and involuntary movements lasting for two hours. His vital signs were as follows: blood pressure of 158/87 mmHg, pulse of 67/min, and temperature of 35.7°C. The temporal artery was not palpable on palpation.

**Figure 1:**
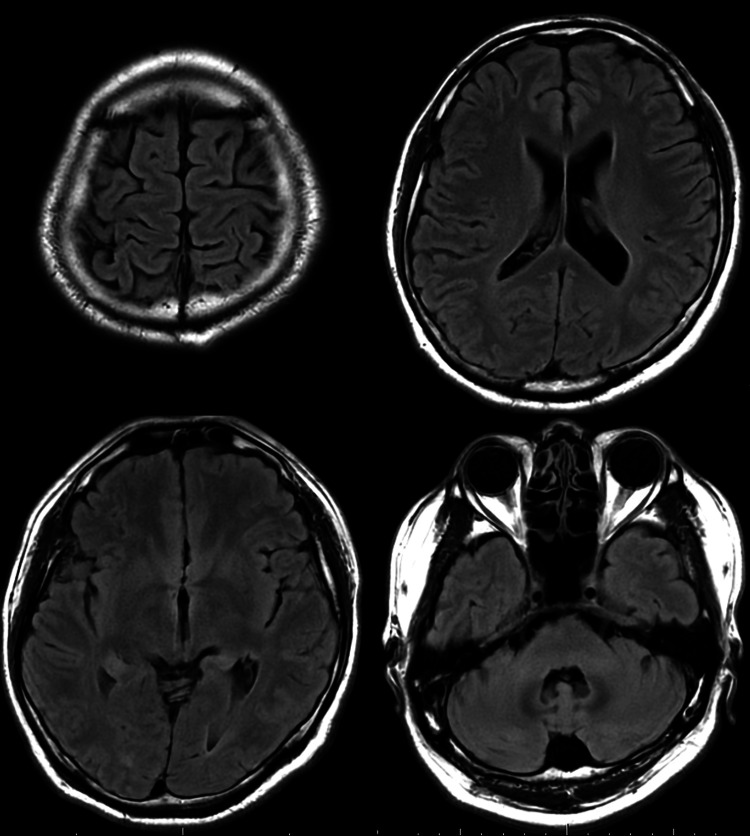
T2-FLAIR brain MRI showing no morphological abnormalities or stroke lesions. T2-FLAIR: T2-weighted fluid-attenuated inversion recovery; MRI: magnetic resonance imaging.

**Figure 2:**
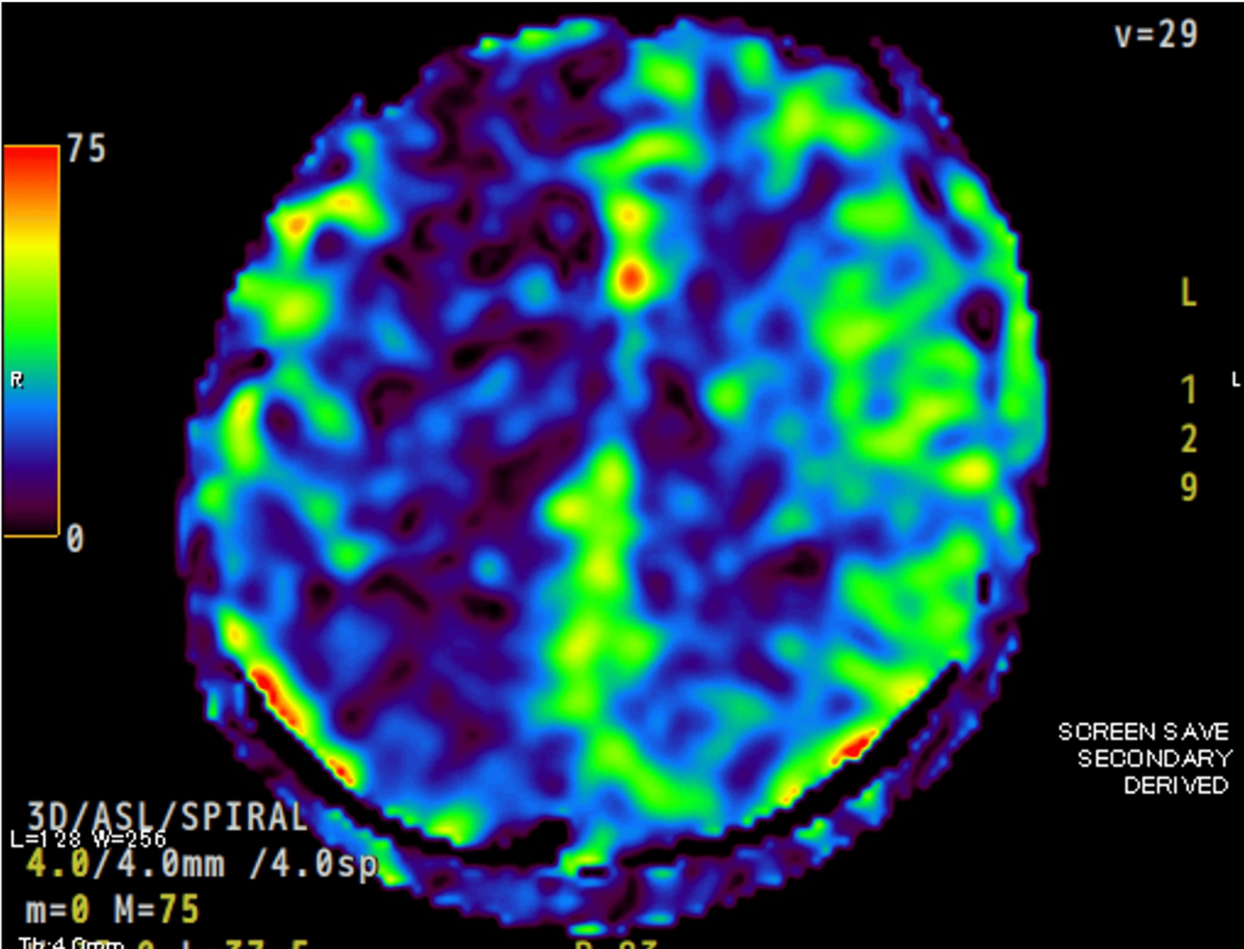
Brain ASL-MRI showing hypoperfusion in the right hemisphere. MRI: magnetic resonance imaging; ASL-MRI: arterial spin labeling MRI.

Neurological examination was pertinent for recurrent, rapid, and twitching contractions of the muscles in the left lower extremity, hyperreflexia in the bilateral patellar tendons, and a positive Babinski sign on the left. Fundoscopy revealed no abnormalities of the optic disc or any retinal abnormalities such as hemorrhages or white spots. Blood tests revealed elevated alanine transaminase of 69 U/L, γ-glutamyltransferase of 127 U/L, and C-reactive protein of 0.27 mg/dL. Cerebrospinal fluid (CSF) analysis showed normal results except for an elevated opening pressure of 30.5 cm CSF. While T2-weighted fluid-attenuated inversion recovery (T2-FLAIR) brain magnetic resonance imaging (MRI) showed no abnormalities (Figure 1), arterial spin labeling MRI (ASL-MRI) revealed decreased blood flow to the right cerebral hemisphere (Figure 2). Magnetic resonance angiography revealed no stenosis in the cerebral vessels. Cervical spine MRI revealed intervertebral disc bulging and foraminal stenosis at C3/4-C5/6, but no signal changes in the spinal cord.

The ophthalmologist assessed the patient with corrected visual acuity of 1.5/1.5 in both eyes, intraocular pressure of 18/19 mmHg, no swelling or atrophy of the optic disc, no abnormal findings such as white spots or hemorrhages in the retina, and no signs suggestive of inflammation. We diagnosed him with migraine and myoclonus and administered 10 mg of diazepam and sumatriptan intravenously, after which the patient's involuntary movements in the left lower extremity ceased, and his headache was mildly relieved. The next day of admission, involuntary movement and visual symptoms disappeared completely. Headache disappeared on day 3 of admission. He was discharged from our hospital on day 6. He declined to return to our hospital for a follow-up appointment, instead promising to come back if his symptoms recurred.

## Discussion

We describe a case of visual symptoms followed by a unilateral throbbing headache and involuntary movement in the left lower extremity. The visual symptoms were similar to an aura, and the involuntary movements were consistent with myoclonus, characterized by intermittent, rapid, rhythmic, and twitching muscle contractions. The involuntary movements and visual symptoms lasted for at least three hours, and the headache resolved within 72 hours. Neurologic examination revealed hyperreflexia in the patellar tendons and pathological positive reflex. CSF findings showed increased opening pressure. ASL-MRI revealed decreased blood flow to the right cerebral hemisphere.

Concerning his headache, he experienced visual symptoms (aura) followed by a unilateral throbbing headache, leading to a diagnosis of migraine with aura, according to the International Classification of Headache Disorders, third edition [2]. Typically, auras develop gradually over five to 20 minutes, last less than an hour, and dissipate as the headache begins [1]. In our case, the aura lasted for several hours, but similarly long-lasting auras (for more than an hour and less than seven days) have been reported in 17.6% of migraine with aura [3]. Other reports described visual symptoms lasted for more than one hour in 14% of auras (n = 158) [1]. Therefore, it is not uncommon for an aura to last for several hours.

In light of the elevated opening pressure of CSF in our case, we considered the possibility of idiopathic intracranial hypertension (IIH). Traditionally, IIH has been viewed as a condition primarily affecting the neuro-ophthalmic axis, characterized by elevated intracranial pressure, headache, and papilledema, with a risk of severe and permanent visual loss and debilitating headaches [4]. However, in our patient, the ophthalmologic examination revealed no abnormalities in the optic disc. Interestingly, although migraine and IIH are distinct disorders, they often coexist and may be interconnected [5]. Additionally, elevated intracranial pressure can occur coincidentally in migraine patients, and normalizing the pressure does not necessarily lead to headache relief [5]. While the exact mechanism is still unclear, existing literature suggests that patients with migraines may experience increased CSF pressure.

In relation to the ASL-MRI findings, our case showed decreased blood flow in the right cerebral hemisphere. ASL-MRI is a technique used to assess cerebral blood flow noninvasively by magnetically labeling inflowing blood [6]. Converging ASL evidence has demonstrated that abnormal CBF, exceeding the boundaries of a single vascular territory, with a biphasic trend dominated by an initial hypoperfusion (during the aura phenomenon but also in the first part of the headache phase) followed by hyperperfusion, characterizes migraine with aura attack and can represent a valuable clinical tool in the differential diagnosis from acute ischemic strokes and epileptic seizures [7]. Another study observed changes in the middle cerebral artery blood flow among migraine patients, finding that 63% experienced no change, 33% had decreased blood flow velocity, and 4% had increased blood flow velocity [8]. This study included patients with both migraine with aura and without aura. Another study examined cerebral blood flow changes by dividing subjects into two groups based on the presence of aura [9]. In patients with migraine with aura, a decrease in velocity in the extracranial segments of the vertebral artery was noted, sometimes combined with vertebral artery hypoplasia and reduced perfusion in the middle cerebral arteries [9]. On the other hand, excessive perfusion in the middle cerebral arteries was the most prominent hemodynamic pattern in patients with migraine without aura [9]. Therefore, we believe that these reports support the ASL-MRI findings in our case.

In our case, a notable feature was the presence of myoclonus in the left lower limb. In the literature, we found reports of myoclonic tremors of the eyelids (blepharoclonus) [10], stapedial myoclonus [11], and drug-induced myoclonus (caused by topiramate or indomethacin) [12,13], all accompanied by migraine headaches. Additionally, some hereditary diseases, including spinocerebellar ataxia, mitochondrial disease, familial hemiplegic migraine, familial cortical myoclonic tremor with epilepsy (FCMTE), and idiopathic basal ganglia calcification, may present with myoclonic epilepsy or myoclonus [12-21]. Although we did not perform genetic testing, the patient had no developmental or growth issues, brain MRI was unremarkable except for cerebral perfusion, and he had no symptoms other than migraines with aura and myoclonus. Furthermore, he was not taking any medication before visiting our hospital. Therefore, it seemed to be different from myoclonus caused by a mechanism that has previously been reported. We suspect that widespread hypoperfusion in the right cerebral cortex, which was related to migraine with aura, may have caused focal myoclonus. Although the exact mechanism in our case remains unclear, given several case reports of migraines accompanied by myoclonus, it is possible that myoclonus is a rare complication of migraine with aura.

There is a major limitation in this report. We diagnosed the involuntary movements as myoclonus based on clinical neurological findings, including intermittent, rapid, rhythmic, and twitching muscle contractions. To confirm the diagnosis of myoclonus, neurophysiological tests such as electromyography (EMG) and electroencephalography (EEG) would be advisable. To rule out familial adult-onset myoclonic epilepsy associated with migraine [22], monitoring in an epilepsy monitoring unit or long-term video EEG monitoring, ideally prolonged for three to five days, would have been necessary.

## Conclusions

We reported a rare case of migraine with aura accompanied by myoclonus. Our case also exhibited increased CSF pressure and decreased blood flow in the right cerebral hemisphere, leading us to conclude that these symptoms could be attributed to the migraine. Although the detailed mechanisms remain unknown, myoclonus may be caused by diffuse hypoperfusion in the cerebral hemisphere, which is related to migraine with aura. Further studies are needed to uncover the underlying mechanisms of myoclonus and migraine.
